# A gradual and sustainable approach to diagnosing sleep and circadian disturbances in dementia

**DOI:** 10.1002/alz.71654

**Published:** 2026-07-20

**Authors:** Biancamaria Guarnieri, Domeniko Hoxhaj, Francesca Buracchi Torresi, Dario Arnaldi, Enrica Bonanni, Matteo Carpi, Riccardo Cremascoli, Claudio Liguori, Gemma Lombardi, Greta Mainieri, Federica Provini, Rosalia Silvestri, Monica Puligheddu, Michelangelo Maestri Tassoni

**Affiliations:** ^1^ Center of Sleep Medicine, Department of Neurology Villa Serena Hospital Città S. Angelo, Pescara Italy; ^2^ Fondazione Villaserena per la Ricerca Città S. Angelo, Pescara Italy; ^3^ Department of Clinical and Experimental Medicine, Neurology Unit University of Pisa Pisa Italy; ^4^ Department of Neuroscience (DINOGMI) University of Genoa Genoa Italy; ^5^ Neurofisiopatologia IRCCS Ospedale Policlinico San Martino Genoa Italy; ^6^ Department of Human Neurosciences Sapienza University of Rome Rome Italy; ^7^ Sleep Medicine Center, Neurology Unit University Hospital of Rome Tor Vergata Rome Italy; ^8^ IRCCS, Istituto Auxologico Italiano Auxologico Villa Caramora Verbania Italy; ^9^ Department of Systems Medicine University of Rome Tor Vergata Rome Italy; ^10^ Neurology Unit IRCCS Neuromed Pozzilli (IS) Italy; ^11^ Neurology 8—Dementias and degenerative diseases of CNS Unit Fondazione IRCCS Istituto Neurologico Carlo Besta Milan Italy; ^12^ IRCCS Istituto delle Scienze Neurologiche di Bologna Bologna Italy; ^13^ Department of Biomedical and NeuroMotor Sciences University of Bologna Bologna Italy; ^14^ Sleep Medicine Center, Department of Clinical and Experimental Medicine University Hospital “G. Martino”, University of Messina Messina Italy; ^15^ Department of Medical Sciences and Public Health University of Cagliari Cagliari Italy

**Keywords:** actigraphy, Alzheimer's disease, biological assessment of circadian disturbances, dementia, home sleep apnea testing, polysomnography, questionnaires, sleep and circadian rhythm disturbances diagnosis, stepped care approach

## Abstract

Evidence linking sleep and circadian disruptions to the course of dementias, particularly Alzheimer's disease, has expanded. Such alterations are detectable from preclinical stages and parallel the disease progression. Assessing and managing sleep and circadian disturbances in patients with dementia remains challenging. New technologies are emerging, but their validation is still pending. We prepared a clinical review outlining a stepped‐care, gradual, and sustainable approach aimed at achieving the most accurate possible diagnosis of different sleep disturbances. This review encompasses diagnostic methods ranging from questionnaires to instrumental assessments, progressing from simpler to more complex techniques including biological evaluations of circadian rhythm alterations. This work reflects a scientific consensus within the “Sleep” study group of the Italian Association for Dementia (SINdem), supported by certified sleep specialists. The document aims to support clinicians in adopting a tailored approach to the evaluation of sleep disturbances in dementia offering a dynamic framework balancing complexity and feasibility.

## Highlights

- Sleep disturbances in dementia impact significantly on healthcare and caregivers.
- Neurobiological mechanisms link sleep disturbances and neurodegeneration.
- A sustainable approach to the diagnosis of sleep disturbances is necessary.
- We propose a dynamic framework encompassing tools between polysomnography and scales.

## 1 BACKGROUND

As people live longer, the risk of developing Alzheimer's disease (AD) and other dementias increases. Though most common among older adults, dementia cannot be considered a normal part of aging. In 2023, nearly 6.9 million Americans aged 65 and older suffered from AD, the most prevalent form of dementia. This number could increase to 13.8 million by 2060, highlighting the urgent need to develop strategies to prevent the disease, slow its progression, and promote early detection and management of physical, psychological, and behavioral conditions associated with it. Moreover, in 2022, over 11 million American family members and other unpaid caregivers dedicated an estimated 18 billion hours to caring for individuals with dementia. The costs for people with AD and other dementias, their families, and society are increasing. Future dementia care programs are crucial for enhancing the quality of life for patients and their caregivers, utilizing multidisciplinary and multi‐professional approaches.^1^ Italy is the second‐oldest country in the world, with nearly a quarter of its population being 65 years or older. Current estimates suggest that nearly 2 million people in Italy live with dementia or mild cognitive impairment (MCI), and 4 million family members and caregivers are involved in supporting and caring for individuals with these cognitive disorders.^2^

Dementia patients often suffer from sleep disturbances that can significantly impact their quality of life and that of their relatives and caregivers.^3^ Clinically significant sleep disturbances in care home residents are linked to higher distress levels among both residents and staff, along with increased use of psychotropic medications.^4^

Notably, sleep disturbances are associated with severe behavioral and psychological symptoms, which can increase caregiver burden, risk of falls, and reliance on resource‐intensive interventions, including specialized nursing care and early institutionalization.^5^ However, relatively few studies have systematically examined the impact of sleep and circadian disturbance on healthcare resource utilization and associated costs in AD and other dementias. In 2021, a retrospective observational study assessing healthcare costs in AD analyzed a cohort of 3500 AD patients with comorbid insomnia and 9884 without insomnia, demonstrating that patients with insomnia exhibited a higher baseline comorbidity burden, increased prevalence of conditions such as depression, and a greater likelihood of hospitalization, emergency room services, and need for skilled nursing care.^6^

According to the 2010 World Alzheimer Report, the costs of informal care account for approximately 55% of the total cost of dementia care.^7^ Moreover, the majority of caregivers are women, who consistently report higher levels of burden compared to male caregivers.^8^ Up to 91% of caregivers report poor sleep quality, and their sleep has been shown to be significantly impaired compared to that of non‐caregiving age‐matched controls.^9^, ^10^, ^11^ In the coming years, the number of caregivers of individuals with dementia is expected to increase substantially, underscoring their importance as a target population for research and interventions aimed at improving sleep and daytime functioning, with the potential to reduce caregiver burden. Considering the bidirectional relationship between patient and caregiver sleep disturbances and behavioral patterns, emerging treatment strategies increasingly adopt dyadic approaches, incorporating non‐pharmacological interventions such as psychoeducation, Cognitive Behavioral Therapy for insomnia (CBT‐I), mindfulness, bright light therapy, structured exposure to natural light, fatigue management, and promotion of physical activity, delivered concurrently to both patients and caregivers.^12^, ^13^

Prevalence data on sleep and circadian disturbances in home‐dwelling individuals with MCI and various types of dementia vary due to different types of sleep disorders and research modalities. Still, it is estimated that between 25% and 90% of people with dementia have one or more sleep disorders. The most frequent disorders are obstructive sleep apnea (OSA), insomnia, restless legs syndrome (RLS), and excessive daytime sleepiness (EDS). A relevant role has been identified for rapid eye movement (REM) sleep behavior disorder (RBD), with lower prevalence in all‐cause dementia, but a selectively higher prevalence in synucleinopathies.^14^, ^15^, ^16^ A 2012 Italian prevalence study conducted in home‐dwelling individuals with well‐characterized diagnosis of MCI, AD and other forms of dementia, including vascular dementia (VaD), frontotemporal dementia (FTD) and Lewy body/Parkinson's disease dementia (LBD/PDD), investigated the occurrence of insomnia, sleep‐disordered breathing (SDB; primarily OSA), RBD, RLS, and EDS. The study demonstrated that nearly 60% of patients exhibited symptoms clinically compatible with SDB, while more than 50% reported EDS and insomnia. Notably, multiple sleep disturbances frequently co‐occur within the same individual, underscoring the importance of comprehensive and multidimensional assessment to guide integrated therapeutic strategies. Overall, sleep disturbances were more prevalent in patients with VaD, FTD and LBD/PDD compared to those with AD.^16^ Patients with VaD showed a higher prevalence of SDB than those with AD, consistent with the well‐established association between SDB in patients with stroke.^17^ Moreover, vascular cognitive impairment (VCI), ranging from MCI to VaD caused by ischemic or hemorrhagic stroke, and cerebrovascular pathology (subclinical white matter lesions and microinfarcts) associated or not with AD or other dementias neuropathology, shares some risks and pathogenetic factors with SDB, OSA in particular, sleep fragmentation, changes in sleep quality and total sleep time (TST).^18^ Several studies confirmed the high prevalence of insomnia, EDS, RBD, RLS in LBD and the higher prevalence of these sleep disturbances in LBD as compared to AD.^19^ A 2022 review on sleep disturbances in LBD confirmed the presence of at least one sleep disturbance (poor sleep quality, EDS, RBD) in 90% of LBD patients.^20^ Regarding OSA, the prevalence has been estimated in 20%–30% of PDD patients and in 18%–35% in LBD patients. To date, few indications and recommendations are available regarding the diagnosis and treatment of sleep disorders in LBD, which are often not accurately diagnosed and managed.^21^

Regarding FTD, the most common form of the disease is the behavioral variant (bv FTD), which accounts for 12%–16% of all primary dementias and is the most common cause of dementia in individuals under 65 years.^22^ The relationship between bvFTD and sleep disturbances has received limited research attention: insomnia, EDS and SDB are common and significantly impair the quality of life of patients and their caregivers. Results based on questionnaires and scales are indicative of a high prevalence of sleep disturbances, whereas the objective assessment of sleep via polysomnography (PSG) has been poorly explored.^23^, ^24^, ^25^

In the past two decades, the evidence on the relationship between sleep and circadian alterations and the trajectory of various forms of dementia, especially AD, has grown exponentially.^26^ Several studies confirmed the role of sleep and sleep–wake rhythm disturbances as independent risk factors for cognitive decline, all‐cause dementia, AD, and vascular dementia.^27^, ^28^, ^29^, ^30^

Sleep and circadian alterations are present from the preclinical and prodromal stages of AD and follow in parallel the progression of the disease.^20^, ^31^, ^32^, ^33^ Several studies, including those carried out with cerebrospinal fluid (CSF) and plasma biomarkers of neurodegeneration, have demonstrated that circadian alterations can be considered not only a risk factor but also a disease marker.^34^ Even in synucleinopathies, circadian alterations accompany the progression of the disease and are present from the very early stages.^35^, ^36^

Furthermore, numerous studies have demonstrated a strong, bidirectional relationship between sleep, circadian disturbances, and neurodegeneration in AD, grounded in specific neurobiological and molecular mechanisms. Sleep‐wake regulation is governed by a bistable “flip‐flop” switch based on reciprocal inhibition between wake‐promoting and sleep‐promoting networks, ensuring rapid and stable transitions between vigilance states.^37^ Wake‐promoting pathways include ascending cholinergic, monoaminergic, histaminergic, and glutamatergic systems, while sleep‐promoting neurons within the ventrolateral and median preoptic nuclei inhibit arousal centers via gamma‐aminobutyric acid‐ergic (GABAergic) and galaninergic transmission.^38^ Orexinergic neurons further stabilize this system by reinforcing wakefulness and preventing inappropriate state transitions, under the modulatory influence of circadian signals from the suprachiasmatic nucleus and homeostatic sleep pressure.^39^ From a neuropathological perspective, tau pathology emerges early in brainstem nuclei such as the locus coeruleus and dorsal raphe, often preceding cortical amyloid deposition and clinical symptoms. These data suggest that the subcortical system is a primary mechanism connected with sleep disturbances since the preclinical stages of AD.^40^, ^41^ Degeneration of these regions disrupts noradrenergic and serotonergic signaling, while loss of orexinergic neurons in the lateral hypothalamus and histaminergic neurons in the tuberomammillary nucleus further impairs wake‐promoting mechanisms, promoting EDS and sleep state instability.^42^, ^43^ In parallel, dysfunction of the suprachiasmatic nucleus leads to circadian desynchronization, contributing to fragmentation of rest‐activity cycles and irregular sleep‐wake patterns.^44^, ^45^

At the electrophysiological level, these alterations manifest as both macro‐ and microarchitectural changes.^46^ PSG studies consistently show reduced TST, sleep efficiency, slow‐wave sleep (SWS), and REM sleep, alongside increased wake after sleep onset,^47^ with similar but milder patterns already detectable in MCI.^48^ More specifically, reduced slow‐wave activity, decreased sleep spindle density, impaired *K*‐complex generation, and disrupted slow oscillation‐spindle coupling reflect thalamocortical dysfunction and impaired sleep‐dependent memory consolidation.^49^, ^50^

Alterations of local sleep oscillations in SWS, spindles and REM sleep have been demonstrated to be connected to different cortical and subcortical areas of neurodegeneration associated with amyloid, tau deposition and neuronal loss.^51^ In addition, REM sleep electroencephalography (EEG) slowing has emerged as a potential early biomarker, particularly in amnestic MCI, where it correlates with cognitive deficits,^52^ and a reduced REM sleep percentage has been associated with basal forebrain atrophy in amnestic MCI patients.^53^

Alterations in sleep microstructure provide additional pathophysiological insight, since the cyclic alternating pattern (CAP), a physiological marker of non‐REM (NREM) sleep fluctuations,^54^ is significantly reduced in MCI and even more in AD, suggesting a loss of the brain's intrinsic ability to generate adaptive oscillations necessary for maintaining sleep continuity and supporting synaptic plasticity. In this framework, CAP alterations may represent an early and sensitive electrophysiological biomarker of neurodegeneration.^54^, ^55^

At the molecular level, some pivotal studies demonstrated that SWS alterations, sleep deprivation and sleep–wake cycle are able to drive brain amyloid and tau dynamics both in humans and animal models.^56^, ^57^ Consistently, dysregulation of sleep architecture, including both SWS and REM sleep, is associated with changes in CSF biomarkers across different stages of AD, reflecting increased accumulation of amyloid‐β and tau in the brain.^58^, ^59^

A key mechanistic pathway underlying these correlations between sleep and neurodegeneration is the glymphatic system,^60^, ^61^, ^62^ which facilitates the clearance of neurotoxic proteins predominantly during deep NREM sleep. Disruption of sleep continuity and slow‐wave activity impairs this clearance process, further promoting neurodegeneration. A 2024 comprehensive review has integrated evidence linking sleep and circadian disturbances with CSF and circulating biomarkers across multiple disorders, including AD, LBD, FTD, and PD, suggesting sleep as a potential modifiable factor toward the prevention of neurodegeneration.^63^

Collectively, these findings support a multilevel model in which sleep disturbances in AD and other dementias arise from the convergence of circuit instability, thalamocortical dysfunction, molecular dysregulation, and progressive neurodegeneration.^46^, ^64^

Building on new insights into sleep's crucial role in the progression of AD and other dementias, an increasing number of neurologists specializing in dementia diagnosis and care are now thoroughly exploring sleep disturbances and habits in individuals with cognitive decline and dementia. This investigation extends from the preclinical phases of the disease and is conducted in close collaboration with sleep experts. Accordingly, sleep biomarkers are emerging as indicators of neurodegeneration, particularly during the preclinical or prodromal stages of cognitive decline.^63^

The assessment and management of sleep and circadian disturbances in dementia patients are often challenging and may appear time‐consuming; however, these processes are critically important and valuable not only to circumvent the use of inappropriate pharmacological interventions but also to explore potential new therapeutic strategies. Additionally, the diagnostic work‐up of sleep disorders improves sleep quality and quantity for both patients with cognitive decline and their caregivers, while also aiding in prevention efforts.

Unfortunately, the availability of instrumental tools to diagnose and monitor sleep disturbances in these patients remains limited, especially since older adults and dementia patients are very numerous and have increased in recent decades.

On the other hand, dementia patients often cannot adequately comply with complex sleep assessments, such as PSG and video‐PSG (VPSG) in sleep laboratories.

In recent decades, new, simplified tools like actigraphy have been increasingly adopted. Actigraphy plays a well‐established role in diagnosing insomnia and circadian rhythm sleep–wake disorders (CRSWDs). Recent studies also show its effectiveness in assessing other sleep disturbances such as narcolepsy and RBD, positioning this device as an important intermediary between clinical history assessments and VPSG, which remains the gold standard for diagnosis.^65^, ^66^, ^67^, ^68^

New technological advancements (contact and noncontact sleep monitoring systems) are rapidly developing to diagnose and manage sleep disorders, but they still need to be widely validated.^69^ They represent a relevant tool for future research agendas in sleep medicine, including dementia patients.

In 2014, the “Sleep” study group of the Italian Association for Dementia adhering to the Italian Neurological Society (SINdem) published the first and, to date, only Italian guidelines on the clinical assessment and management of sleep disorders in individuals with MCI and dementia, which were approved by the Italian Neurological Society (SIN).^70^

“Poor sleep results in an increased risk of morbidities and mortality in demented patients and is a source of stress for caregivers” is the opening statement of these guidelines, which hold significant clinical and social implications. To date, growing evidence continues to support these claims, and some progress has been made to reduce the severe effects of sleep disturbances on disease progression, caregiver stress, high social costs for assistance, and the need for hospitalization.

The 2024 Italian Ministry of Health and the Italian National Institute of Health (Istituto Superiore di Sanità ISS) guidelines on the diagnosis and treatment of dementia and MCI adopted the following recommendation regarding the management of non‐cognitive symptoms: “For people living with dementia who have sleep problems, consider a personalized multicomponent sleep management approach that includes sleep hygiene education, exposure to daylight, exercise, and personalized activities”. Moreover, the Italian guidelines underline the relevant diagnostic role of RBD in dementia with Lewy bodies (LBD).^2^

In 2020, the fifth Canadian consensus conference on the diagnosis and treatment of dementia provided some recommendations for sleep, to help prevent dementia development: “A careful sleep history, including assessment of sleep time, insomnia, daytime sleepiness, napping, and RBD, may facilitate identification of preclinical dementia, or a high risk of developing dementia, and should be included in assessments in both primary care and specialized memory clinic settings 1A (91%). Objective assessment of sleep using actigraphy or PSG can help identify individuals at high risk of developing dementia”. Additional recommendations regarding the reduction of dementia risk were also shared. “Patients suspected of having sleep apnea should be referred for PSG and/or sleep specialist consultation to evaluate treatment options 1C (96%). Avoiding severe sleep deprivation (< 5 hours) and aiming for 7–8 hours of sleep per night may improve cognition and decrease the risk of dementia 1C (94%).”^71^

Building on previous considerations, the current SINdem “Sleep” study group has prepared a clinical review of a “stepped care,” gradual, sustainable approach to obtain the most accurate possible diagnosis of each type of sleep disturbance in people with cognitive decline. This includes selecting appropriate patients who may be candidates for more complex gold standard diagnostics, such as attended PSG and VPSG.

The present work is not designed to provide formal recommendations, but rather to reflect a scientific consensus among dementia and sleep specialists.

This document comprises five chapters that cover diagnostic methods, ranging from questionnaires and scales to instrumental support, progressing from simpler to more complex options, as well as biological evaluations of circadian rhythm alterations. Moreover, the paper features synthetic graphics and tables outlining possible step‐by‐step approaches for assessing sleep and circadian changes based on dementia types and stages, starting from the preclinical and prodromal phases. Throughout the manuscript, particular attention has been devoted to sleep disturbances from the perspective of dementia prevention.

The present document was designed, developed and discussed within the SINdem “Sleep” study group. Finally, a comprehensive revision was undertaken with the support of additional sleep‐certified specialists, culminating in a document that is valuable for clinical practice and, potentially, for research applications.

## 2 SUBJECTIVE EVALUATION OF SLEEP–WAKE HABITS, SLEEP QUALITY, AND DISORDERS: SCALES AND QUESTIONNAIRES

A thorough patient sleep history is an essential part of the comprehensive clinical assessment.

Several scales and questionnaires have been developed to evaluate sleep, circadian habits, and sleep quality, aid in screening for sleep disorders, measure their severity, and assist in differential diagnosis. As a general limitation, the correspondence between objective and subjective sleep measurements is often difficult to establish, and for many symptoms and variables it may be unavailable. In elderly and cognitively impaired subjects, this problem (and gap) is even more relevant, and the discrepancy between subjective and objective sleep measures is well documented in patients with MCI and AD, as well as the oldest old who tend to overestimate their sleep duration and quality, even if conflicting data exist.^72^, ^73^, ^74^ Scales and questionnaires often require input from the bed partner or, at minimum, from relatives living with the patient. In cases of dementia, the involvement of the primary caregiver, depending on the severity of the disease, is almost invariably necessary.

Moreover, several well‐known, widespread, and standardized sleep questionnaires and scales validated in young and middle‐aged adults are not suitable for older adults and individuals with dementia. To date, no clear and detailed recommendations have been published based on dementia type and severity.

In this paper, we reviewed various scales and questionnaires designed to evaluate overall sleep quality, identify common sleep symptoms and disorders linked to dementia or its preclinical stages—such as insomnia, OSA syndrome (OSAS), RLS, RBD, and EDS—and to assess chronotype in clinical settings. We also referenced other validated scales employed in international clinical research trials. Our focus included widely used questionnaires and scales validated in multiple languages, currently used in people with dementia, with particular attention to their limitations in this population. Table 1, ^75^ provides a summary of this, offering suggestions and proposals for employing questionnaires and scales according to the level of cognition. This document is primarily intended for the Italian population. It should be noted, however, that not all questionnaires and scales have undergone validation in Italy, nor have they been tested in Italian patients with dementia. The corresponding limitations are detailed in Table 1.^75^

**TABLE 1:** Questionnaires and scales to assess sleep disturbances and primary sleep disorders (EDS, insomnia, circadian disturbances, OSA, RLS, RBD) based on cognition level, evaluated with the Clinical Dementia Rating (CDR),^75^ and tailored to the Italian population.

Questionnaire/scale	Administration	Italian validation	Utility	Suggestions/Proposals of applicability according to CDR
PSQI (Pittsburgh Sleep Quality Index)^76^ ^,^ ^77^	Self (informant admitted)	Yes (also in dementia)	Assessment of sleep quality.	CDR ≤ 2
NPI (Neuropsychiatric inventory)^78^ SDI^79^	Informant	No Italian translation (NPI): available^80^	Assessment of sleep disturbances and agitation	CDR ≥ 1
ESS (Epworth Sleepiness Scale)^81^ ^,^ ^82^	Self (informant admitted)	Yes	Assessment of EDS	CDR ≤ 2
EQAS (Essener Questionnaire)^83^	Informant	No Italian translation: available (method of translation described in the text)	Assessment of EDS	CDR ≥ 1
ISI (Insomnia Severity Index)^84^	Self	Yes	Assessment of the perceived severity of insomnia	CDR ≤ 1
SCS (Sleep Continuity Scale)^85^	Self/informant	Yes	Assessment of sleep discontinuity/fragmentation in AD	CDR ≤ 2
MEQ and r‐ MEQ^86^	Self	Yes (only in adolescents) Italian translation: available^87^	Chronotype evaluation	CDR ≤ 1
The sundowning evaluation questionnaire^88^	Informant	Italian version is the original one	Screening and characterization of sundowning (it does not investigate sleep)	No limitation based on dementia severity
Berlin questionnaire^89^	Self (informant admitted)	No Italian translation: available^90^	OSA risk assessment	CDR ≤ 2
Stop Bang questionnaire (complete and short version)^91^	Self (informant admitted)	No Italian translation: available^92^	OSA risk assessment	CDR ≤ 2
Five diagnostic criteria for RLS^93^	Self	International criteria commonly applied in the Italian language	Assessment of RLS symptoms for a clinical diagnosis of RLS	CDR ≤ 2
“Behavioral criteria” for the clinical diagnosis of RLS in dementia^93^	Informant (at the best, the bed partner)	International criteria commonly applied in the Italian language	Assessment of RLS discomfort by a third person for a diagnosis of clinically possible RLS in dementia	CDR ≥ 2
RBD1Q (RBD single‐question screen)^94^	Self participation by spouses/caregivers encouraged	No Italian translation: available^94^	Single question focusing on dream enactment, useful in primary care settings for quick screening in idiopathic /isolated RBD	CDR ≤ 1
RBDSQ (REM sleep behavior disorder screening questionnaire)^95^ ^,^ ^96^	Self	Yes	Multiple‐choice tool for screening RBD	CDR ≤ 0.5
MSQ (Mayo Sleep Questionnaire)^97^	Informant (preferably the bed partner)	No Italian translation: available^98^	Diagnosis of clinically possible RBD	No limitation based on dementia severity

Abbreviations: EDS, excessive daytime sleepiness; OSA, obstructive sleep apnea; RLS, restless legs syndrome.

### 2.1 General sleep habits and quality

**The Neuropsychiatric Inventory (NPI)** has been extensively utilized in research on neuropsychiatric symptoms associated with neurodegenerative diseases for the past 25 years. It is a semi‐structured interview conducted by a clinician with a caregiver of a dementia patient. The inventory can include 10 items, excluding sleep and vegetative disturbances, or 12 items if those are included. If a “screening” question about each specific disturbance is answered positively, subsequent sub questions are then asked. The NPI score is determined from single frequency and severity ratings of the overall behavioral area during the past 4 weeks, rather than from individual sub question ratings.^78^, ^80^

**The Sleep Disorders Inventory (SDI)** was derived from the NPI by using its seven sub questions related to sleep disturbance and maintains the same scoring method. It assesses the frequency, severity, and caregiver burden of sleep‐disturbed behaviors over a defined period before administration. The SDI was validated with actigraph‐based sleep measurements and two weeks of sleep quality ratings (SQR) recorded in a diary by caregivers.^79^

**The Pittsburgh Sleep Quality Index (PSQI)** is one of the most widely used scales to assess sleep quality,^76^ consisting of 19 questions that examine various aspects of sleep over the past 30 days. A score above 5 distinguishes between “good” and “poor” sleepers with high sensitivity and specificity.^99^, ^100^ Questions cover seven scoring areas (subjective sleep quality, latency, duration, habitual sleep efficiency, sleep disturbances, use of hypnotic drugs, and daytime dysfunction), with each component scored from 0 to 3 points. The scale presents high internal consistency and homogeneity. A factor scoring model based on three different domains (sleep efficiency, perceived sleep quality, and daily disturbances) has been tested in older persons.^101^, ^102^ The Italian version of PSQI has been validated.^77^ The PSQI possesses inherent limitations as a self‐report measure, exhibiting diminished reliability in cases of moderate to severe dementia and among the elderly, especially considering known discrepancies between subjectively and objectively measured sleep.^103^ Notably, it includes five items completed by caregivers that are excluded from the total score. The scale is predominantly focused on sleep patterns and habits and does not comprehensively address certain sleep disorders and conditions, such as bedtime motor issues (including akinesia, dystonia, or chorea), RLS, and RBD.^104^ The scale has nevertheless been extensively employed across various psychiatric and neurological conditions, such as dementia, and among older adults for whom the contribution of the primary caregiver is required.

### 2.2 EDS

The **Epworth Sleepiness Scale (ESS)** is the most commonly used self‐report instrument to assess daytime sleepiness.^81^ It is structured into eight questions that assess subjective sleepiness over the past two weeks related to everyday situations in which the patient might feel drowsy or fall asleep. The total score ranges from 0 to 24, with a score above 10 indicating the likely presence of EDS. An Italian version of the ESS was validated and published in 2003 by Vignatelli and colleagues.^82^ Awareness of its limitations is necessary even among the general population, due to significant variability in test‐retest validity, the potential multidimensionality of the scale, and overlap between normal and pathological values.^105^ In cognitively impaired patients, some items, like sleepiness while driving,^106^ are unclear or inapplicable. Subjective perception is further complicated because it may be influenced by the dementia itself,^83^, ^107^ requiring flexibility and sensitivity to the patient's needs.^108^, ^109^ Despite these challenges, the ESS has been widely used in MCI and various dementia types with good reliability up to the early stages of the disease, usually based on informant reports.

In an effort to better investigate EDS in older and dementia patients, other scales and questionnaires have been proposed.

In 2021 Gronewold and collaborators introduced an **adapted version of ESS** that replaced two items and used an interview format, resulting in fewer missing responses and higher internal consistency than the original. However, these results have not been replicated.^110^

The **Essener Questionnaire of Age and Sleepiness (EQAS)** was developed by Frohnhofen H. et al.^83^ and translated into Italian by Manni et al. using the “backward forward translation” method approved by the author (unpublished). The caregiver is asked to report the frequency, intensity, and duration of daytime sleep (morning and afternoon), as well as whether the person sleeps while sitting during the day, including an assessment of the sleep's intensity. The overall score ranges between 0 and 48, with a higher score indicating more severe sleepiness. The questionnaire is appropriate for reliably assessing EDS in older subjects, regardless of their cognitive or communicative abilities.

### 2.3 Insomnia

The **Insomnia Severity Index (ISI)** ^84^, ^111^ is a brief and easy‐to‐administer, widely used scale to assess the severity of insomnia, monitor changes in symptoms over time, and evaluate the effectiveness of therapeutic interventions.^84^ It consists of seven questions that measure the perceived severity of insomnia, its impact on daytime functioning, and the degree of associated concern. Each question has a response scale from 0 to 4, and the total score can range from 0 to 28, with higher scores indicating greater severity of insomnia. The ISI is practical for both clinical and research settings, and an Italian version has been validated.^112^ It has been validated in various age groups and cultural contexts, increasing its utility as a global assessment tool.^113^, ^114^ The scale relies on the patient's personal perceptions, which can be influenced by various psychological and cultural factors;^115^ this could be particularly relevant in patients with mild to moderate dementia. It has been used in some studies on frontotemporal dementia (FTD),^116^ AD, and LBD.^117^ Further research is needed to explore and validate the use of the ISI within this specific context since the scale demonstrates high internal consistency but also exhibits a high level of heterogeneity.^118^

The **Sleep Continuity Scale (SCS)** was designed to evaluate sleep discontinuity and fragmentation, aiming to provide a quick and easy‐to‐use tool for early detection and ongoing monitoring of sleep disturbances in AD. The questionnaire's global score has been associated with measures of cognitive, functional, and behavioral impairment and consists of 9 items assessing sleep discontinuity and fragmentation, displaying high internal consistency.^119^

### 2.4 Circadian orientations and alterations

To assess the subject's circadian orientation, or chronotype, both the **Morningness‐Eveningness Questionnaire (MEQ)** and its **short form (r‐MEQ)** can be used.^86^ The questionnaire facilitates the differentiation between serotine, morning, and intermediate chronotypes. The Italian version is available and has been validated in Italian adolescents.^87^ Its questions are aimed at exploring the optimal times for waking up and going to bed individually, especially when people are free to plan their day and evening. They also examine how tired a person feels within the first half hour after waking, the time of day when they feel at their best, and whether they feel more active in the morning or evening. It is important to note that these questionnaires assess circadian orientation rather than directly measuring the subjects' circadian biological rhythms. They have been used in some relevant studies, but have not been validated in dementia.^120^ However, given the strong link between chronotype, physical activity, and dementia risk in older adults, and the importance of sleep timing during day and night in managing sleep and circadian disruptions throughout dementia progression, it is crucial to assess specific circadian habits and changes from the preclinical or prodromal stages of the disease.

Sundown Syndrome (SS) in dementia patients, characterized by the appearance or worsening of neuropsychiatric symptoms in late afternoon or evening, was described in 1941 by Cameron. It is highly prevalent, but its origin is still debated and uncertain. Its multifactorial pathophysiology involves a circadian dysfunction, primarily affecting the hypothalamic suprachiasmatic nucleus (SCN), the human brain's master clock that regulates circadian rhythms.^121^ Building on earlier reference instruments such as the **1989 Cohen‐Mansfield Agitation Inventory**, Toccacelli Blasi and colleagues developed a new **Sundowning Evaluation Questionnaire** to assess this behavioral disturbance in individuals with dementia. A screening question assesses the presence of behavioral changes during the late afternoon or evening, referring to the past month. The subsequent questions focus on characterizing sundowning by evaluating specific disturbances such as delusions, hallucinations, aggression or agitation, sadness, irritability, happiness without clear reasons, fear of being alone, decreased interest or indifference, apathy, mood fluctuations, eating disorders, and repetitive purposeless actions. The questionnaire does not investigate sleep and circadian alterations.^88^ In 2018, Sevilla et al. demonstrated that age, a higher degree of cognitive impairment, and the presence of insomnia and/or hypersomnia were independent factors predicting the onset of SS in patients with dementia.^122^

### 2.5 OSA

The prevalence of OSA in patients with cognitive deficits is high, estimated at around 40%–60% depending on the methods used.^16^ Early detection of OSA risk can aid diagnosis and treatment, potentially improving health, cognitive abilities, and quality of life, while also possibly delaying neurodegeneration in dementia patients.^123^ Unfortunately, the screening tools currently available appear to have some limitations when applied in populations affected by cognitive decline.^124^, ^125^

The most commonly used screening tools for OSA are the **STOP‐BANG and Berlin questionnaires**.

The **STOP‐BANG Questionnaire (SBQ)** is a concise and simple self‐administered tool, covering four symptom‐related questions and demographic information.^91^ The acronym STOP‐BANG stands for Snoring, Tiredness, Observed Apnea, High Blood Pressure, Body Mass Index, Age, Neck Circumference, and Gender. All assessed variables are dichotomized, and high risk for OSA is defined as positive responses to three or more items. It was translated into Italian by Braido et al. in 2014.^126^ In the general population, the SBQ has excellent sensitivity, but low specificity; it shows high positive and negative predictive values^92^ and has been validated in several clinical settings.^127^ In memory clinics, the effectiveness of the SBQ depends on the accuracy of the information provided, often requiring caregiver involvement.^125^ A 2025 study^128^ found that while the SBQ may be unsuitable to detect moderate or severe OSA in older adults with cognitive impairment, oximetry may be a viable screening tool.

The Berlin Questionnaire on sleep apnea^89^ was translated into Italian by Gassino et al. in 2005.^90^ It identifies high‐risk individuals based on positive responses in at least two of three domains (snoring, daytime sleepiness, hypertension/body mass index [BMI]). Meta‐analysis test characteristics depend on setting, location, OSA diagnostic cutoff, and OSA prevalence. A meta‐analysis demonstrated a reasonable sensitivity of 79% (Apnea–Hypopnea Index [AHI] ≥ 5/h) and 82% (AHI ≥ 15/h) but exhibited low specificity ranging from 32% to 53% (AHI ≥ 5/h) and 35% to 39% (AHI ≥ 15/h) within a sleep clinic population,^129^ suggesting this questionnaire may be a useful screening tool. However, a lower sensitivity compared to the SBQ in the general population has also been reported.^130^ In subjects with cognitive decline, it has been used as a screening tool in patients with MCI and mild to moderate AD,^16^ but symptoms such as fatigue, apathy, cognitive impairment, and other sleep disturbances may overlap with symptoms of OSAS, thereby complicating the interpretation of the questionnaire.

### 2.6 RLS

The diagnosis of RLS is primarily clinical and relies on positive answers to the essential diagnostic criteria developed and published by the International RLS Study Group in 2003 and revised in 2014.^93^ These criteria include: (1) an urge to move the legs usually, but not always, accompanied by, or felt to be caused by, uncomfortable and unpleasant sensations in the legs; (2) the urge to move the legs and any accompanying unpleasant sensations that begin or worsen during periods of rest or inactivity, such as lying down or sitting; (3) the urge to move the legs and any accompanying unpleasant sensations are partially or totally relieved by movement, such as walking or stretching, at least as long as the activity continues; (4) the urge to move the legs and any accompanying unpleasant sensations during rest or inactivity only occur or are worse in the evening or at night than during the day; (5) the occurrences of the above features are not solely attributed to symptoms primary to another medical or behavioral condition (e.g., myalgia, venous stasis, leg edema, arthritis, leg cramps, positional discomfort, habitual foot tapping). Other supportive criteria include the presence of periodic limb movements, response to dopaminergic treatment, family medical history, and the absence of daytime sleepiness. The 2003 criteria have been used in MCI and mild‐to‐moderate dementia patients, including the 2012 Italian multicenter study on the prevalence of sleep disturbances in MCI and dementia.^16^

Furthermore, in 2003, specific, primarily behavioral criteria for RLS in more cognitively impaired elderly were published. The criteria are: (1) signs of leg discomfort such as rubbing or kneading the legs and groaning while holding the lower extremities; (2) excessive motor activity in the lower extremities such as pacing, fidgeting, repetitive kicking, tossing and turning in bed, slapping the legs on the mattress, cycling movements of the lower limbs, repetitive foot tapping, rubbing the feet together, and the inability to remain seated; (3) signs of leg discomfort are exclusively present or worsen during periods of rest or inactivity; (4) signs of leg discomfort are diminished with activity; (5) criteria 1 and 2 occur only in the evening or at night or are worse at those times than during the day. All five criteria are stated as necessary for diagnosis.

The specificity and sensitivity of standardized questionnaires for RLS have been extensively criticized (see Fulda et al., 2021 for a review).^131^ However, a single question for rapid screening of RLS in neurological clinical practice has been developed and published, and it aligns with more comprehensive questionnaires.^132^

In 2015, a Behavioral Indicators Test–Restless Legs (BIT‐RL) was developed using two indicators of leg movement and 13 indicators of excessive motor activity in the lower extremities,^133^ and it was subsequently used to diagnose RLS.^134^ However, in most studies on RLS and dementia, the diagnosis of RLS was made through a clinical interview with a sleep specialist.^16^, ^135^

The International RLS Severity Scale (IRLS) was created by the International RLS Study Group, using questions contributed by its members. It consists of 10 questions rated from 0 to 4 and has been validated through face‐to‐face patient interviews. It is the primary tool used to assess RLS severity in both pharmaceutical and nonpharmaceutical studies of therapeutic agents for RLS and has been translated into several languages, including Italian.

### 2.7 RBD

According to the International Classification of Sleep Disorders (ICSD‐3‐text revision), the diagnosis of RBD requires clinical and VPSG criteria.^136^ Since its occurrence in or before neurodegenerative diseases, particularly in synucleinopathies,^137^ several questionnaires have been introduced and validated to assess the presence of possible or probable RBD.^67^, ^138^

The **REM Sleep Behavior Disorder Screening Questionnaire (RBDSQ)** ^95^ is a self‐administered, multiple‐choice tool designed to assess the frequency and severity of dream enactment behaviors and associated features. It is valuable for its quick administration, has shown high sensitivity in clinical settings, and has been validated in Italian.^96^

**The Mayo Sleep Questionnaire (MSQ)**, developed by the Mayo Clinic, is often administered to a bed partner or a close family member, providing a unique third‐person perspective on the patient's sleep behavior.^97^ It is particularly effective in identifying behaviors that the patient might not be aware of. This tool has been validated in an aging and dementia cohort and translated into Italian.^98^

The **Innsbruck REM Sleep Behavior Disorder Inventory (RBD‐I‐5)** is a five‐item tool designed to screen for RBD.^139^ The first part inquires about five aspects of RBD symptoms: violent or aggressive dream content, sleep‐related vocalizations, sleep‐related extensive movements, sleep‐related injurious behaviors, and dream‐behavior isomorphism. The second part aims to assess the frequency of these symptoms over the past year.^139^

**The RBD Single‐Question Screen (RBD1Q)** consists of a single item addressing dream enactment behavior, making it particularly useful for rapid screening in primary care settings. Designed for self‐administration, it also encourages input from spouses or caregivers. However, its simplicity may limit its ability to detect more subtle cases of RBD. Among screening tools, the RBD‐I‐5 demonstrated the best overall diagnostic performance in terms of sensitivity, specificity, and positive predictive value, whereas the RBD1Q achieved the highest specificity. The RBD1Q has been translated into several languages, including Italian, by native‐speaking medical translators or RBD experts fluent in both English and the target language. The Italian version is available in the *Supporting Materials* of the 2012 paper by Postuma et al.^94^

Stefani et al.^138^ examined RBDSQ, RBD1Q, and RBD‐I‐5 in a recent study involving 400 patients consecutively referred to a sleep center for the first time. The research confirmed earlier findings, showing that these tools have limited specificity, accuracy, and positive predictive value. Nevertheless, the authors propose that these questionnaires primarily serve as screening tools to exclude idiopathic or isolated RBD—cases lacking evident neurodegenerative features—given their high sensitivity and negative predictive value. Interpretation becomes more challenging in the context of neurodegenerative disorders, as data from patients with LBD and Parkinson's disease (PD) are more complex to interpret.

It is important to reiterate that a confirmed diagnosis of RBD must be based on VPSG and should not be mistaken for a diagnosis of possible or probable RBD, which is only clinically determined. A possible algorithm might involve using RBD1Q as an initial screening tool, followed by RBDSQ or MSQ as additional steps, and then referring suspected cases to sleep specialists for more detailed instrumental investigations.^67^, ^140^

The **International RBD Severity Scale (IRBD‐SSS)** has recently been validated as a new tool developed by the International RBD Study Group to assess the overall severity of RBD symptoms in the home setting. Two versions of the IRBD‐SSS exist: one for the patient (IRBD‐SSS‐PT) and another for the bedpartner. Both consist of three components—vocalizations, body movements, and injury—with a fourth component in the patient version that assesses dream content. Both the patient and bedpartner versions of the IRBD‐SSS show excellent acceptability, good internal consistency and external validity, and high reproducibility, making the IRBD‐SSS a valuable tool for evaluating the severity of RBD symptoms in clinical settings and trials.^141^

### 2.8 Sleep diaries

Sleep diaries are useful for assessing the subjective features of insomnia and circadian rhythm disturbances, as well as for guiding behavioral interventions. They are inexpensive and can be easily completed in natural sleeping environments, including nursing homes and clinical settings. In individuals with MCI and dementia, sleep diaries offer a practical and low‐cost method for monitoring sleep–wake patterns and disturbances; however, their reliability may be affected by cognitive deficits. Several studies have reported discrepancies between subjective (diary‐based) and objective (actigraphy or EEG) sleep measures, particularly as cognitive decline advances. Despite these limitations, sleep diaries remain a valuable source of information, especially when used alongside objective assessment tools.^142^, ^143^, ^144^

## 3 ACTIGRAPHY

### 3.1 Overview of actigraphy and sleep–wake cycle recording

Over the last four decades, actigraphy has become a crucial assessment tool in sleep research and sleep medicine.^66^, ^145^, ^146^, ^147^ Actigraphy measures shifts in activity levels using data obtained from small, watch‐like portable devices. These devices, called actigraphs, consist of a triaxial accelerometer that quantifies movement exceeding a certain threshold, typically complemented by additional sensors, including a photodiode to record light exposure, a temperature sensor to identify device removal, and an event‐marker button that the subject can press to signal specific events (e.g., lights‐off, wake‐up time, diurnal naps, and drug intake), depending on the recording targets.

Typically worn on the nondominant wrist for extended periods (such as 1 or 2 weeks), the actigraph monitors rest‐activity cycles in real‐life settings. Its primary benefit over standard ambulatory sleep tests with PSG is the ability to gather ecological data, allowing assessment of the variability of sleep–wake patterns over time within the person's usual environment.

Sleep–wake and circadian metrics are obtained from raw activity scores through scoring algorithms, which are typically developed for a specific commercial device and tested through validation studies.^147^ Actigraphy variables can be broadly categorized into three groups: sleep timing parameters, sleep continuity parameters, and circadian rhythm measures. Despite some variability across devices, sleep timing measures usually include bedtime, wake‐up time, midpoint of night sleep, and total time in bed (TIB). Sleep continuity measures comprise actigraphy‐estimated conventional sleep parameters, such as TST, sleep onset latency (SOL), wake after sleep onset (WASO), sleep efficiency (SE, i.e., TST/TIB*100), the number of awakenings, the duration of the longest sleep episode, and the amount of motor activity during sleep (estimated in arbitrary, device‐dependent units). Circadian measures are classified into parametric and non‐parametric measures. Parametric measures mainly rely on the cosinor method, which fits a cosine function to rest‐activity data. This method uses the periodic nature of the cosine function to extract parameters describing the circadian rhythm, such as period, amplitude (i.e., the height of oscillations), MESOR (rhythm‐adjusted mean), and acrophase (i.e., the time of maximum peak within the cycle). Nonparametric measures have been devised to account for the aspects of the rest‐activity rhythm not adequately modeled by a cosine function (i.e., those not associated with the parameters of a known mathematical function).^148^, ^149^, ^150^, ^151^ These variables summarize key features of circadian rhythms, including intradaily variability (IV), which measures rhythm fragmentation within a day; interdaily stability (IS), which assesses rhythm synchronization as day‐to‐day similarity of activity patterns; the mean activity level during the least active 5 hours (L5) and the most active 10 hours (M10); and their ratio, which represents the relative amplitude (RA) of the rhythm.

Estimates of sleep duration and latency obtained with actigraphy have shown good concordance with PSG,^152^ and standardized procedures for actigraphy monitoring in research and clinical settings have been established.^153^

### 3.2 Actigraphy use in sleep disorders

Actigraphy is currently recommended for the assessment and monitoring of several sleep‐related conditions, including circadian rhythm sleep–wake disorders and shift‐work disorder. It is also utilized for characterizing sleep–wake patterns and sleep continuity within populations that require specific assessments (e.g., older adults) and among individuals with insomnia, insufficient sleep syndrome, or central disorders of hypersomnolence.^66^, ^152^, ^154^, ^155^ In the evaluation of sleep‐disordered breathing with home sleep apnea testing (HSAT), the concomitant use of actigraphy to assess TST might also improve diagnostic accuracy for identifying respiratory events.^152^ However, standalone actigraphy is not sufficient for diagnosing OSA.^156^

Although actigraphy is not recommended for the clinical diagnosis of periodic limb movements of sleep (PLMS),^152^ actigraphic devices worn on the ankles can help identify PLMS, with increasing evidence showing good accuracy compared to PSG for both single and bilateral actigraphs.^157^, ^158^, ^159^

Focusing specifically on insomnia, recent studies synthesized by Liguori et al.^66^ indicate that actigraphy effectively distinguishes patients with insomnia from good sleepers and can monitor sleep changes resulting from effective treatment.

Regarding RBD, patients consistently show increased daytime naps, disrupted sleep–wake cycles, and decreased daytime motor activity, as measured by actigraphy. Quantitative analysis of actigraphy data has also been proposed as a preliminary screening tool for detecting isolated RBD.^65^, ^66^, ^68^, ^160^, ^161^ This evidence is particularly relevant for the early diagnosis of α‐synucleinopathies. Since RBD is frequently a prodromal manifestation of these neurodegenerative diseases, early recognition is highly warranted.^162^, ^163^, ^164^ The potential of actigraphy markers to identify individuals with RBD who may develop full‐blown α‐synucleinopathies should be further investigated. A single prospective study reported that RBD patients who converted to an α‐synucleinopathy within 2 years exhibited a higher nap frequency and lower daytime activity levels compared to RBD patients who did not develop neurodegenerative diseases in the same time span.^165^

### 3.3 Actigraphy use in neurodegenerative disorders

Given the growing body of research highlighting the relevant role of circadian rhythms in neurodegenerative disorders such as AD and PD,^166^, ^167^, ^168^ actigraphy might be an appropriate methodology for assessing sleep–wake patterns in these disorders and other conditions leading to cognitive decline and dementia. It offers the advantage of monitoring patients over a prolonged period, especially when acquiring a PSG recording may be challenging. Specifically, beyond clinical sleep disorders, symptomatic AD patients typically present with disrupted sleep–wake rhythms, increased rhythm fragmentation, and a characteristic phase delay that diverges from the phase advance typically reported in physiological aging.^31^, ^169^ In fact, circadian and sleep–wake alterations have been shown to manifest early in the course of the disease and to play a role in modulating the levels of brain neuropathology.^64^, ^166^ Specifically, the well‐known phenomenon of sundowning—characterized by an exacerbation of cognitive and behavioral symptoms in the evening—is recognized as a significant clinical feature in the management of patients with dementia, potentially leading to sleep fragmentation and insomnia.^170^

Notably, non‐parametric actigraphy variables were initially employed to characterize patients with AD,^149^ and alterations in rest‐activity rhythms have recently been shown to discriminate both patients with dementia due to AD and those with MCI from healthy controls.^171^, ^172^ Similarly, phase delay, reduced rhythm amplitude, and increased nighttime activity have been reported in patients with PD.^173^, ^174^

Moreover, large epidemiological studies have demonstrated that actigraphy measurements of rest‐activity rhythms can predict long‐term cognitive decline, potentially aiding early diagnosis and the identification of modifiable risk factors for neurodegenerative disorders.^175^, ^176^, ^177^, ^178^ A recent study also showed that fragmented sleep–wake patterns (i.e., higher IV), assessed through one‐week actigraphic monitoring, predicted subsequent amyloid‐β positron emission tomography (PET) burden in community‐dwelling adults without dementia, with a stronger association in participants with an apolipoprotein E ε4 genotype, a known risk factor for AD.^34^

Although these results were obtained with heterogeneous research protocols and exhibit some inconsistencies,^179^ overall, they indicate the potential of actigraphy in population screening for dementia and neurodegeneration.

### 3.4 Actigraphy use in Alzheimer's disease and dementia research

In summary, actigraphy provides a suitable solution for evaluating the efficacy of specific treatments on sleep and circadian endpoints in elderly people with neurodegenerative diseases, permitting ecological assessment of sleep patterns and capturing fine‐grained aspects of circadian rhythms. In patients with AD, actigraphy has been used to assess the effectiveness of the dual orexin receptor antagonist lemborexant in the treatment of irregular sleep–wake rhythm disorder, highlighting specific effects on circadian‐related parameters such as RA and L5, which could not have been detected with other methodologies.^180^ Additionally, for treatments that directly impact circadian rhythms, thorough evaluation of actigraphic outcomes may help clarify the controversial results regarding the effectiveness of light therapy in improving sleep quality in elderly patients with dementia.^181^ Actigraphy‐derived parameters, such as the time of light exposure, could also help tailor exogenous melatonin treatment^182^ and assess its purported sleep‐enhancing and neuroprotective effects in patients with cognitive impairment due to AD and PD.^183^, ^184^

Finally, future research is warranted to explore the potential of actigraphy for monitoring and dynamically assessing behavioral manifestations of AD, such as agitation and sundowning, whose occurrence, intensity, and circadian variation may be linked to rest‐activity rhythm parameters.^185^ In this context, integrated light sensors may also prove useful for recording the amount of light exposure, thereby preventing and mitigating behavioral and psychological symptoms associated with sundowning.^186^ Furthermore, the extended application of actigraphy in real‐world environments can assist in evaluating treatment outcomes by monitoring not only sleep patterns but also daytime activity measures related to movement and the comprehensive sleep–wake cycle rhythm.

## 4 Home Sleep Apnea Testing (HSAT)

HSAT is becoming increasingly valuable for diagnosing respiratory sleep disorders in dementia patients. Its relative simplicity, ease of use, and patient tolerance make it a practical choice in this population, where traditional PSG may be less feasible.

**FIGURE 1:**
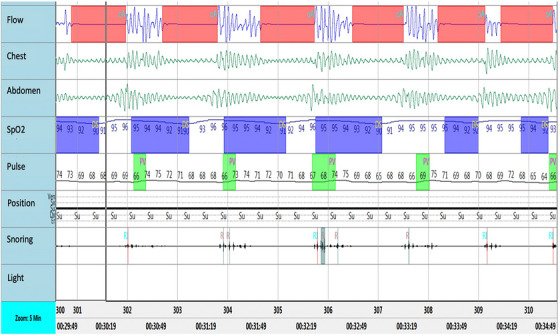
The figure illustrates examples of obstructive apneas using HSAT. The red‐highlighted areas in the flow signal indicate periods of complete airflow cessation, characteristic of obstructive apneas. Simultaneously, the thoracic and abdominal effort bands (green traces) show ongoing respiratory effort, reflecting the attempt to overcome the airway obstruction. At the end of each apnea, there is a transient increase in thoraco‐abdominal effort and a subsequent recovery in oxygen saturation (SpO2), as shown by the blue band, marking the resolution of the apnea. HSAT, home sleep apnea testing.

HSAT generally includes several key components: a respiratory effort band around the chest and abdomen, a nasal cannula with a pressure transducer to detect airflow and snoring vibrations, and an oximeter to monitor pulse and oxygen saturation, or alternatively, peripheral arterial tonometry (PAT) with oximetry and actigraphy (Figure 1).^187^ A PAT‐based system is worn like a wristwatch, use a finger‐mounted pneumatic sensor to detect changes in digital arterial volume with each heartbeat. These changes reflect dynamic fluctuations in peripheral vascular resistance driven by sympathetic nervous system activation during respiratory disturbances and arousals. The integration of the PAT signal with pulse oximetry, heart rate variability, and actigraphic data facilitates the automated identification of obstructive events, eliminating the need for nasal cannulas or thoracoabdominal inductive belts. Several studies have demonstrated a strong correlation between PAT and PSG in detecting moderate to severe OSA, even in special populations such as those with chronic obstructive pulmonary disease, heart failure, and Down syndrome. Additionally, PAT can estimate TST and stages, offering greater comfort and feasibility for patients who struggle with in‐laboratory PSG or traditional HSAT setups.^188^ Other advanced devices may incorporate photoplethysmography, which uses infrared light to measure volumetric blood variations, providing sleep staging and arousal data even without EEG monitoring.^189^

HSAT is typically designated for patients at high risk of moderate to severe OSA, as indicated by the presence of EDS and at least two of the following three criteria: habitual loud snoring; observed apnea, gasping, or choking episodes; or diagnosed hypertension. The risk of OSA is also stratified using questionnaires, such as the Berlin Questionnaire^89^ and the SBQ,^91^ though recent findings suggest that the latter may be unsuitable for detecting moderate or severe OSA in older adults with cognitive impairment. Moreover, a 2024 study proposed oximetry as a viable screening tool in memory clinics.^128^ Highlighting the importance of employing objective variables for large‐scale population screening, a multistep machine learning approach was documented for wrist‐worn smartbands, trained with actigraphic and photoplethysmographic data. This algorithm demonstrated the ability to stratify the severity of OSA with results comparable to the SBQ, but with the advantage of using objective data.^190^

The diagnostic accuracy of HSAT diminishes in patients with mild OSA, who are more effectively diagnosed by the PSG‐derived AHI, which measures the number of respiratory events per hour of TST.^191^ However, compared to PSG, these polygraphic (PG) methods are more accessible and have been more broadly validated for diagnosing and treating OSA.

HSAT must be conducted under the supervision of accredited sleep centers and board‐certified sleep medicine physicians. Each HSAT recording is conducted over at least one night. A technically adequate diagnostic test requires a minimum of 4 h of sufficient oximetry and flow data, obtained during a recording session that encompasses the patient's habitual sleep period. To enhance the analysis of HSAT data, supplemental information can be provided by sleep diaries, wherein the patient, or their caregiver, should note the periods of WASO and the subjective TST for the night of the examination.^192^ Despite its limitations, such as the potential underestimation of mild OSA and the inability to detect hypopnea associated with cortical arousals, HSAT remains a reliable tool for initial diagnosis and monitoring. It provides the Respiratory Event Index (REI), which measures the number of apneas and hypopneas per hour of monitoring time (Figure 1).^187^ This index is essential for classifying the severity of OSA and guiding treatment choices. There is a tendency for REI to underestimate AHI, though this does not always impact diagnostic decisions. REI is always less than or equal to the AHI obtained through PSG.^191^

HSAT can also help identify OSA phenotypes because, even if the REM phase cannot be detected with this method, clusters of phasic desaturations occurring approximately every 90 minutes can indicate events during REM. These are typical of REM‐related OSA, which is more prevalent in women.^193^, ^194^, ^195^ Additionally, a REI in the non‐supine position that is less than 50% of that in the supine position indicates the presence of positional OSA (POSA).^196^

Patients suffering from dementia often face challenges with traditional sleep studies due to cognitive impairments, thereby making HSAT a more viable option. Research has demonstrated the feasibility of HSAT in individuals with cognitive impairment, excluding severe cases (MoCA < 10). In a study by Colelli et al.,^197^ an 86% success rate was reported, attributed to comprehensive instructions and participant support, including in‐person demonstrations and access to a helpline. However, these findings may not extend to patients with severe cognitive impairment, for whom the use of HSAT remains challenging and should be considered on a case‐by‐case basis, taking into account the availability of caregiver assistance.

HSAT's technical simplicity and patient‐friendly nature make it particularly suitable for deployment within domestic environments. The ability to conduct tests in familiar settings can improve sleep quality and data accuracy, as patients are more likely to follow their usual routines. This consideration is particularly pertinent for dementia patients, who may experience anxiety or disorientation in clinical settings. Its simplicity fosters patient compliance and reduces the likelihood of technical issues. Additionally, the cost‐efficiency of HSAT in comparison to in‐laboratory PSG, in conjunction with its validation against the latter,^198^ underscores its suitability for widespread screening and monitoring.

While HSAT offers numerous benefits, it is not without limitations. The absence of EEG monitoring can lead to an underestimation of the REI, especially in women who often experience arousals without desaturations.^195^, ^199^ Furthermore, factors such as skin pigmentation can affect oximetry accuracy,^200^ with darker‐skinned individuals facing a higher risk of having their desaturations underestimated by oximetry. Other factors such as thick skin, poor circulation, and nail polish, can also impact oximeter readings. HSAT is not recommended for patients at high risk of central sleep apnea, obesity hypoventilation syndrome, comorbid pulmonary conditions such as chronic obstructive pulmonary disease, neuromuscular disorders, and non‐respiratory sleep disorders, including parasomnias.^192^ Moreover, HSAT's focus on cardiorespiratory parameters means it cannot assess RBD or PLMS, which are crucial components of comprehensive sleep evaluations. Nevertheless, when PSG or VPSG are unavailable or unfeasible due to compliance issues, HSAT is particularly useful to rule out OSAS, the most common mimic of RBD, or to address its potential comorbidity with RBD. This is critical, as untreated OSAS may coexist with RBD and complicate the evaluation and management of symptoms. Accordingly, a stepwise approach is recommended, starting with HSAT, and in case of inconclusive data or a high suspicion of other sleep disorders, an in‐laboratory or a home‐VPSG should be conducted to confirm the diagnosis and inform treatment decisions.^67^

## 5 PSG and Video recordings

### 5.1 Hospital (laboratory) PSG and VPSG

PSG is a recording procedure that includes, along with EEG, electromyogram (EMG), and electrooculogram (EOG), the addition of simultaneous multichannel tracings from respiratory, cardiac, and limb muscle activity.^201^ EEG, EOG, EMG, and electrocardiogram (ECG) are recorded by applying surface electrodes to the patient. Respiratory activity is measured by means of thermal sensors and/or nasal air pressure transducers, piezoelectric or inductance plethysmography belts, and oximetry, in accordance with the American Academy of Sleep Medicine (AASM) guidelines. EEG electrode positions are determined by the International 10–20 System. At a minimum, frontal (F3–F4), central (C3–C4), and occipital (O1–O2) derivations are required to stage sleep. These derivations are referenced to the contralateral mastoid processes (M1 and M2).^187^ The recommended EOG derivations and electrode placements include one electrode placed 1 cm below and 1 cm lateral to the left outer canthus of the left eye (E1), and another placed 1 cm above and 1 cm lateral to the right outer canthus of the right eye (E2). The recommended derivations are E1–M2 and E2–M2. To record chin EMG, three electrodes should be positioned as follows: one at the midline 1 cm above the inferior edge of the mandible (Chin Z), one 2 cm below the inferior edge of the mandible and 2 cm to the right of the midline (Chin2), and one 2 cm below the inferior edge of the mandible and 2 cm to the left of the midline (Chin1). The standard chin EMG derivation comprises either of the electrodes positioned below the mandible, referenced to the electrode situated above it. The other inferior electrode serves as a backup in case one of the primary electrodes malfunctions.^187^ EEG montage may be adjusted based on clinical suspicion, such as using a standard sleep montage for suspected RBD, or a complete EEG montage for suspected confusional arousals from NREM sleep, nocturnal seizures, or hallucinations. In addition to chin and anterior tibialis EMG, supplementary muscles may be employed and tailored to specific suspicions (e.g., wrist flexor/extensor in cases of suspected RBD). Furthermore, depending on the patient's cooperation and suspected diagnosis, recordings may be conducted over one or two nights.

VPSG requires a synchronized video and audio recording along with polysomnographic parameters. PSG and VPSG may be conducted in a sleep laboratory or alternative settings such as a hospital ward or at the patient's residence. If a sleep technician is actively present and able to intervene during the procedure, the exam is classified as attended; if not, it is considered unattended. The advantages of an attended VPSG include the ability to interrogate or test the patient directly, such as during sleep parasomnia episodes or epileptic seizures, as well as generating external triggers like acoustic stimuli. It also allows for quick resolution of technical artifacts and can be tailored for specific video recording needs, such as framing adjustments or zooming in on particular details.

Attended VPSG is the most accurate objective sleep assessment for most sleep disorders and is essential for the definitive diagnosis of some, including parasomnias, as outlined by the ICSD.^136^, ^202^ Opting for an attended VPSG is essential when assessing patients with diverse medical and sleep‐related conditions, such as SDB, movement disorders, and parasomnias. This approach is particularly important in complex cases or when patients are on medications that influence sleep.^70^

### 5.2 Home PSG and VPSG

While attended PSG and VPSG remain the gold standards for diagnosing complex nocturnal sleep disorders, they are expensive, time‐consuming, and often involve long waiting periods. These procedures can also be poorly tolerated by patients with dementia, as they are conducted in unnatural environments that may be difficult for certain groups, such as those with cognitive impairments, to accept. Additionally, there is a low likelihood of recording sufficiently structured sleep of adequate duration or capturing at least one typical episode during one or two VPSG assessments. For these reasons, alternative recording tools such as home PSG and VPSG have been developed. In this context, the presence of a reliable and dedicated caregiver is imperative for the successful completion of the exam, which may otherwise be unfeasible for certain patients, especially those with cognitive impairments.

Home PSG and VPSG require a specialized sleep technician to provide the patient with the same equipment used in the sleep lab, as well as a synchronized video camera upon request. Home PSG can be utilized to evaluate sleep stages, potential sleep‐related EEG paroxysmal activities, sleep‐related breathing disorders, PLMS, and the presence or absence of REM sleep without atonia.^203^ Home VPSG requires the patient or their caregiver to correctly position and activate the camera and infrared light. The camera should be placed at a frontal location at the bottom of the bed, elevated to provide a clear view of the entire patient's body. Additional considerations include sleeping uncovered to facilitate the semeiological analysis of nocturnal behaviors and wearing dark clothing, since white garments can increase reflection in video recordings. This type of exam might be deemed more acceptable than laboratory VPSG, as the patient is free to move within their usual environment, which may enhance the chances of capturing typical nocturnal episodes. Another advantage is that the patient is not bound by the technician's schedule and can maintain their normal sleep–wake habits, allowing for the recording of episodes that may occur in the morning hours.

If the procedure is executed properly and typical episodes are documented, home VPSG could be diagnostic, as the synchronized PG parameters enable the characterization of sleep, including the sleep stage from which the episodes originate, as well as other parameters such as muscle tone, electrocardiographic activity, or respiration. A well‐trained caregiver might also be able to question the patient during a typical episode. Conversely, a technical problem might not be resolved quickly and could invalidate the exam.

### 5.3 Home video

Although home VPSG may guarantee a greater quantity of nocturnal recordings relative to attended or unattended sleep laboratory VPSG, the number of recordings is still limited to a few nights and it may not adequately capture the episodes documented in the clinical history, especially if they occur infrequently. In addition, home VPSG might be unfeasible in particular cases, such as when patients do not cooperate, lack a reliable caregiver, or are unable to manage sleep equipment. Consequently, it may be more practical to rely solely on home‐video monitoring, without the implementation of any PSG setup. Home videos recorded with professional high‐quality infrared motion‐detector cameras can now capture and store consecutive episodes over multiple nights. Homemade video recordings have proven useful in diagnosing nocturnal episodes of different etiologies.^204^, ^205^, ^206^, ^207^ They offer multiple advantages: wide availability, low costs, high acceptability and feasibility, the capacity to record patients in their regular sleep environment, and to conduct repeated recordings. This increases the likelihood of capturing multiple, longer, and more complex episodes compared to the standard in‐laboratory setting, while also allowing comparison of the episodes’ semeiology.^204^, ^205^, ^206^, ^207^ Of course, a conclusive diagnostic assessment cannot rely solely on home‐video recordings; a definitive diagnosis requires VPSG. Nonetheless, under the guidance of sleep experts, home recordings can serve as additional support and complement clinical history, especially for patients where collecting a detailed medical history is challenging. Finally, home recordings may be a valuable follow‐up tool once a definitive diagnosis is made, helping to monitor the frequency of actual episodes or response to treatment.^208^ However, these devices are not yet available for clinical use and are limited to research settings. Therefore, electromedical companies should be encouraged to develop home‐recording devices that comply with the General Data Protection Regulation.

### 5.4 Wearable EEG systems

Recent advancements in the development of low‐cost single‐channel EEG headbands have opened new possibilities in health monitoring and brain–computer interface (BCI) systems, with promising prospects also in dementia research and management.

A fast and accurate analysis of sleep architecture, along with measurements of slow wave and REM sleep, may serve as valuable biomarkers of cognitive reserve in dementia patients. In fact, research has shown that TST, SE, and slow wave activity all predict cognitive decline following a nonlinear, inverse “U‐shaped” pattern, indicating that sleep measures have an optimal intermediate range where cognitive scores remain stable.^209^, ^210^, ^211^

To date, four distinct categories of wearable EEG systems have been developed: (a) rigid headbands, (b) flexible headbands, (c) highly flexible EEG sleep‐monitoring systems, and (d) ear–EEG sleep‐monitoring plugs and patches.^212^ These systems can acquire different types of physiological activities, ranging from those that record only brain activity via EEG, primarily from frontal derivations,^212^, ^213^, ^214^ to those that also record movement, position, respiration, and heart rate using a 3D accelerometer and a pulse oximeter.^215^ They have been validated by comparing their automated sleep scoring with PSG‐based scoring, revealing a high level of agreement for most of them,^212^ with the highest agreement observed in a wireless sleep‐monitoring patch system.^216^ Studies assessing two nights of sleep revealed that a single night's recording was sufficient to characterize certain sleep activities, such as abnormal slow wave sleep, sleep spindle activity, and heart rate variability. However, conducting assessments over two nights improved the evaluation of other sleep biomarkers.^217^ In addition, research comparing various wearable systems found that rigid headbands may disrupt sleep more than flexible variants, reducing their suitability for individuals with sleep disorders.^218^ Nevertheless, the results obtained from wearable EEG systems should be interpreted with caution, as they have predominantly been validated on small samples of adult subjects,^212^ with only a limited number of studies involving larger elderly populations.^219^, ^220^ In summary, while wearable single‐channel EEG systems show promise, they require further validation across large populations, including healthy individuals, patients with various sleep disorders, and those experiencing cognitive decline.

Furthermore, sleep assessment is increasingly being extended by emerging contact and non‐contact monitoring technologies, available as both consumer‐ and research‐grade solutions.^69^, ^221^ These include multisensor wearables combining accelerometry with physiological signals such as photoplethysmography, as well as nearable and airable systems that collect information from the sleep environment, for example through mattress‐based or audio‐based sensing.^221^, ^222^, ^223^, ^224^, ^225^ Such tools show promise for the longitudinal, ecological assessment of sleep‐wake patterns, but most consumer devices still lack sufficient validation for clinical use, and evidence in older adults and people with dementia remains limited.^69^, ^142^, ^144^ In these populations, validation is particularly challenging because cognitive impairment, behavioral symptoms, altered sleep architecture, and reliance on caregivers may affect both feasibility and measurement accuracy.^142^, ^144^, ^221^ Accordingly, these technologies represent an important area for future sleep medicine research, including evaluation in dementia.

## 6 Biological Assessment of Circadian Disturbances

Circadian rhythms are fundamental biological processes that govern various physiological functions, including sleep–wake cycles, hormone secretion, and body temperature regulation. Disruptions in these rhythms can have profound effects on health and cognition. Normal aging in humans is associated with changes in circadian rhythms, including phase advancement and disruption of the sleep–wake cycle, characterized by increased nocturnal awakenings and daytime napping, reduced circadian amplitudes of hormone secretion, and fluctuations in core body temperature (CBT). Seniors, due to greater isolation, are less exposed to zeitgebers and external cues, which accentuates these phenomena. These disruptions are further exacerbated in neurodegenerative disorders, with patients often exhibiting irregular sleep–wake rhythm disorder and sleep–wake phase disorders.^166^

The underlying mechanisms of circadian dysfunction in neurodegeneration include reduced light exposure, degeneration of the retina and optic nerve, and impairment of the central circadian pacemaker, namely the SCN of the anterior hypothalamus. Melatonin disruption, due to its neuroprotective and anti‐inflammatory properties, may also play a role in neurodegenerative pathology.^226^

Emerging evidence suggests bidirectional relationships between circadian homeostasis, sleep disorders, and neurodegeneration, indicating that circadian dysfunction might play a significant role in the progression of neurodegenerative dementias.^227^, ^228^

The pattern of an individual's circadian rhythm can be measured with both biological and behavioral markers. Landmark experiments by Czeisler et al.^229^ identified CBT, as well as melatonin and cortisol secretions, as circadian biomarkers, the oscillations of which are regulated by the SCN.

### 6.1 Melatonin

Melatonin is a hormone primarily produced by the pineal gland in response to darkness. The accumulated findings suggest that the onset of nocturnal melatonin secretion is synchronized with the opening of the nocturnal sleep gate, thus helping regulate the circadian rhythm of sleep propensity.^230^ Melatonin rhythms are often disrupted in individuals with sleep disorders and neurodegenerative diseases.^227^ Nassan and Videnovic^228^ discussed how circadian disturbances, including anomalies in melatonin secretion, are prevalent in diseases like AD and PD. Furthermore, the onset of melatonin secretion in dim light conditions, known as the dim light melatonin onset (DLMO), tends to be delayed in subjects with early‐stage AD, whereas their subjective sleep parameters and chronotype appear comparable to those of healthy controls.^231^ These disruptions can exacerbate disease symptoms and progression, making melatonin a potential target for therapeutic interventions aimed at stabilizing circadian rhythms in these populations.

Melatonin levels can be measured in plasma, urine, and saliva.^232^ The circadian rhythm of melatonin in saliva or plasma is among the most frequently employed biomarkers for assessing the circadian phase in humans.^233^, ^234^ Circulating melatonin levels are often preferred as a circadian marker because they remain relatively stable despite various external influences. For example, while excessive carbohydrate intake can produce significant changes in CBT and heart rate, melatonin concentration remains essentially uninfluenced by this factor.^234^

DLMO is a reliable marker for assessing circadian phase position.^235^ Pullman et al. (2012)^236^ validated an at‐home method for DLMO assessment (Table 1). This offers a feasible approach for continuous monitoring of circadian rhythms outside of clinical settings, reducing the need for frequent laboratory visits, and enhancing patient comfort.

It has been recommended that in studies using melatonin as a circadian phase marker, exposure to dim light should be initiated 1–2 hours before the earliest melatonin onset.^235^, ^237^ This implies that dim light should start at 5 p.m. and blood sampling should begin around 6 p.m. The recommended illumination level for sampling is currently 10 lx. Absolute darkness appears to have no advantage over dim light in reducing the suppressant effect of bright light on melatonin production. The plasma levels of the major melatonin metabolite, aMT6S, have also been employed to measure DLMO.^238^ The fact that melatonin onset is not affected by biochemical and physiological confounding factors accounts for its comparatively higher reliability in measuring circadian phase position.^235^, ^237^ However, individual differences may still need to be taken into account. Human plasma melatonin levels during the day are usually below 10 pg/mL, while at night, they typically exceed 40 pg/mL. In low melatonin producers, plasma levels are significantly lower. Therefore, a 2 pg/mL plasma threshold is often recommended for measuring DLMO.^235^ Another option is to employ the procedure recommended by Voultsios et al.^239^ for determining a threshold, which involves calculating the mean of three points and adding twice their standard deviation. Leibenluft et al.^240^ employed salivary samples to measure DLMO and proposed this measurement as the most practical and reliable method to assess the circadian phase. Further studies have contributed to the rapid adoption of salivary testing as the preferred sampling method for melatonin, as it is relatively non‐invasive and more acceptable to patients. An additional advantage of this method is that, with proper training, patients can collect samples at home and deliver them to the laboratory for testing. In recent studies, salivary sampling has emerged as the primary method for assessing physiological melatonin levels, especially in longitudinal investigations requiring repeated measurements. It should be noted that melatonin levels and, consequently, DLMO measured in a sleep laboratory might differ from those obtained in a natural setting, such as at home.^241^, ^242^, ^243^ Recent studies have further validated home‐based protocols for DLMO, demonstrating good feasibility and concordance with laboratory measures even in real‐life settings.^244^, ^245^ In adults, DLMO is defined as the time when salivary melatonin reaches 4 pg/mL,^246^, ^247^, ^248^, ^249^, ^250^ and is considered normal if it occurs between 7.30 p.m. and 10 p.m.^248^, ^251^

DLMO is widely used to diagnose and treat CRSWDs. For example, individuals with delayed sleep–wake phase disorder (DSWPD) often exhibit a delayed DLMO, indicating a shift in their circadian phase.^246^ Properly timed melatonin supplementation can help realign the circadian phase in such individuals.^248^ Studies have also indicated that the timing of DLMO can predict the optimal window for light therapy and melatonin administration to treat CRSWDs.^252^, ^253^ Furthermore, DLMO timing has been correlated with subjective sleep quality and mood, reinforcing its utility in clinical settings.^254^

### 6.2 Body temperature

Body temperature typically fluctuates over the 24‐hour cycle, reaching its lowest point (nadir) during the early morning hours and peaking in the late afternoon to early evening.

The nadir of CBT generally occurs a few hours before the habitual wake time, when sleep propensity is highest.^255^ This drop in body temperature is associated with the release of melatonin, which facilitates sleep onset. Conversely, the rise in body temperature toward the end of the sleep period helps promote wakefulness and alertness. Vitiello et al.^256^ highlighted that young adults typically exhibit a robust circadian temperature rhythm, while older adults may experience blunted amplitude and shifted timing of these rhythms.

The relationship between body temperature and sleep is bidirectional. Not only does the circadian regulation of body temperature influence sleep timing and quality, but sleep itself can also affect thermoregulation. Body temperature rhythms are also linked to cognitive performance and alertness.

Advancements in wearable technology, especially actigraphy devices, and artificial intelligence (AI) offer exciting possibilities for continuous and non‐invasive monitoring of body temperature.^257^ These technologies can provide real‐time data on circadian phase and identify subtle shifts in temperature rhythms that may indicate circadian misalignment. AI algorithms can analyze these data to predict optimal times for therapeutic interventions, thereby enhancing the precision and effectiveness of treatments for CRSWDs.^233^ The circadian rhythm of peripheral skin temperature has been shown to correlate significantly with sleep quality, suggesting that targeted modulation of skin temperature may serve as a chronobiological intervention for sleep problems.

### 6.3 Cortisol

The secretion of cortisol follows a diurnal pattern regulated by the SCN. Cortisol levels typically peak in the early morning just before waking, known as the cortisol awakening response (CAR). This peak promotes wakefulness and prepares the body for the demands of the day. Throughout the day, cortisol levels gradually decline, reaching their lowest point in the late evening, facilitating sleep onset.^258^

Continuous, noninvasive measurement of cortisol levels using wearable sensors can provide detailed circadian profiles, helping to identify disruptions and guide personalized interventions. AI algorithms can analyze these datasets to optimize the scheduling of therapeutic interventions, such as pharmacological treatments or behavioral therapies, thereby enhancing their effectiveness.^228^

### 6.4 Future directions

Within a few years, new methodologies will be applied to the circadian processes, in particular AI and transcriptome‐based biomarkers.

AI algorithms can process continuous data on melatonin levels, body temperature, and cortisol rhythms to create comprehensive circadian profiles,^221^ and AI‐driven insights can optimize personalized light therapy schedules and melatonin timing. Klerman et al.^233^ compared the variability of different circadian markers and suggested that AI could enhance the precision of these measurements.

Over the past decade, blood transcriptome‐based biomarkers have emerged as promising indicators of the human circadian phase.^259^ The discovery that the clock gene network operates in nearly all tissues, including peripheral blood mononuclear cells (PBMCs), suggests the existence of cell‐autonomous clocks and offers an alternative approach for circadian assessment. Integrating machine learning with large‐scale transcriptomic data offers new opportunities to characterize individual circadian rhythms, advancing the goals of precision medicine.^260^ Several methods have been proposed; notably, “time signature” emerges as a promising machine‐learning approach to predict physiological time based on gene expression in human blood.^261^

## 7 FINAL DESCRIPTIVE SUMMARY OF A GRADUAL, SUSTAINABLE APPROACH TO DIAGNOSE SLEEP AND CIRCADIAN DISTURBANCES IN DEMENTIA

This final section presents a structured, step‐by‐step approach for assessing sleep and circadian disturbances in individuals with dementia, progressing from simpler to more advanced methods according to dementia severity.

**FIGURE 2:**
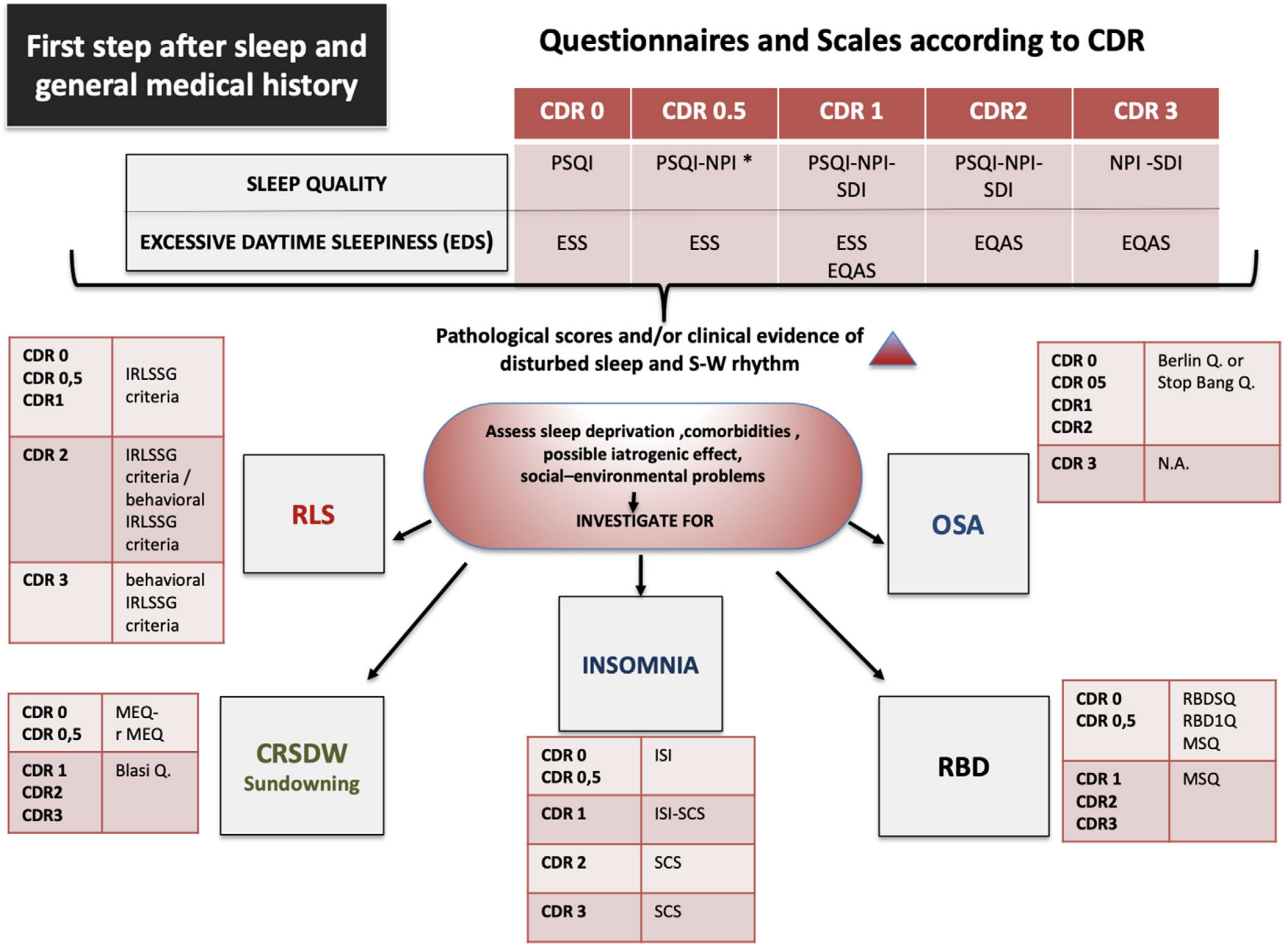
Assessment of sleep and circadian disturbances with scales and questionnaires selected according to dementia severity. CDR, Clinical Dementia Rating (0–3); CRSWD, circadian rhythm sleep–wake disorders; EQAS Essener Questionnaire of Age and Sleepiness; ESS, Epworth Sleepiness Scale; IRLSSG Criteria, International RLS Study Group Criteria; ISI, Insomnia Severity Index; MEQ, Morningness‐Eveningness Questionnaire; MSQ, Mayo Sleep Questionnaire. NPI, Neuropsychiatric Inventory; OSA, obstructive sleep apnea; PSQI, Pittsburgh Sleep Quality Index; r‐MEQ, reduced‐Morningness‐Eveningness Questionnaire; RLS, restless legs syndrome; RBD, rapid eye movement sleep behavior disorder; RBD1Q, RBD Single Question; RBDSQ, RBD Screening Questionnaire; SCS, Sleep Continuity Scale; SDI, Sleep Disturbances Inventory. *Not available.

**FIGURE 3:**
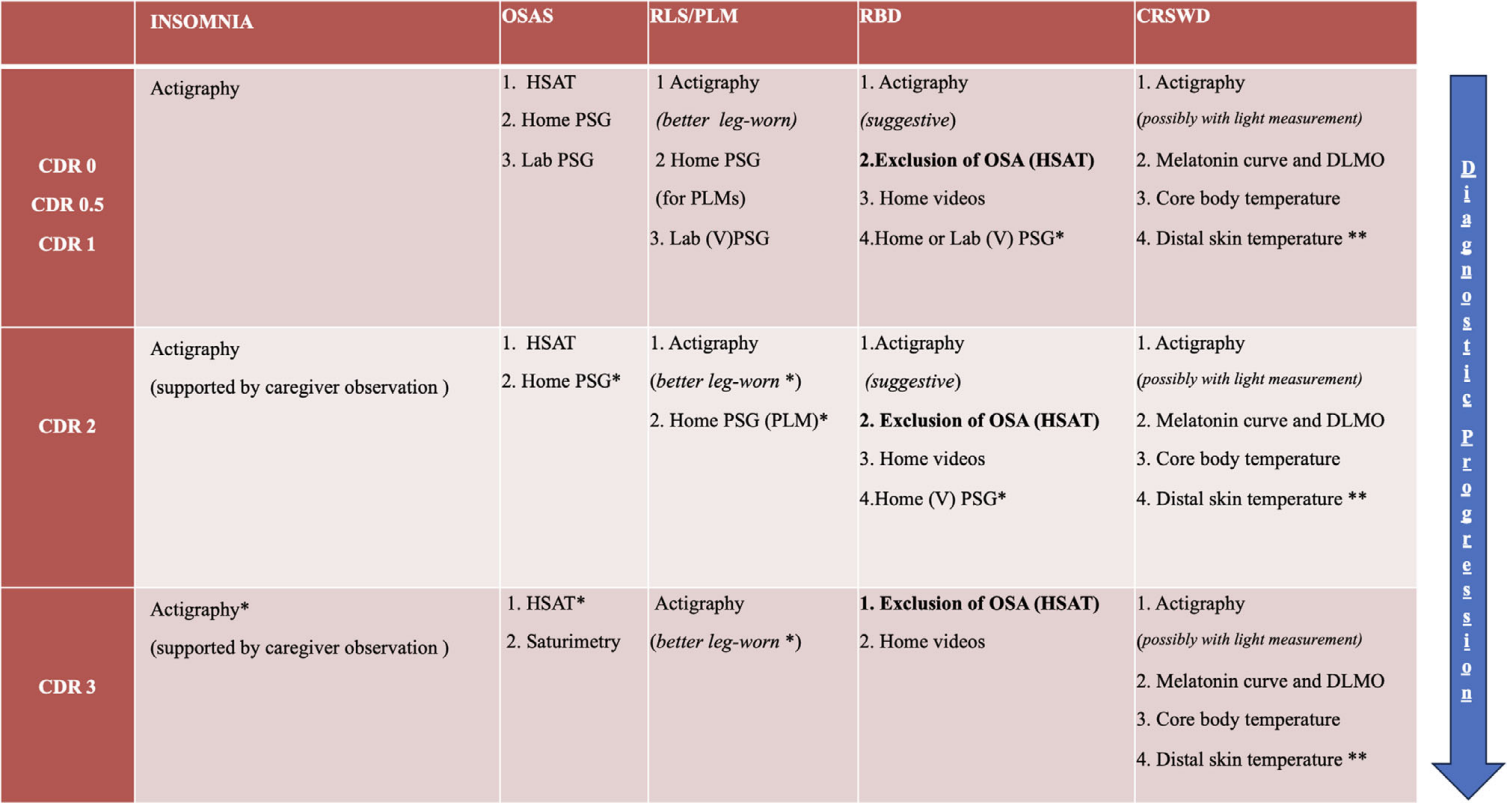
Stepwise progression in the use of instrumental diagnostic tools, as needed. The progressive use of instrumental diagnostic tools should cease once an adequate diagnosis has been established. For every primary sleep disorder, the diagnostic process should also explore possible comorbid sleep conditions. Multiple and differential diagnoses should be systematically evaluated in each patient. * Depending on patient cooperation. ** Wearable temperature sensors. CDR, Clinical Dementia Rating (0–3); CRSWD, circadian rhythm sleep–wake disorders; DLMO, dim light melatonin onset; HSAT, home sleep apnea testing; (V)(Home) PSG, (video) (home) polysom, nography; RLS/PLM, restless legs syndrome/periodic limb movements; RBD, rapid eye movement sleep behavior disorder.

We provide a summary diagram and corresponding figures (Figure 2, Figure 3) outlining a practical and sustainable strategy for achieving the most accurate assessment of sleep and circadian alterations. The progression of instrumental assessments is intended to stop once an adequate diagnostic conclusion is reached.

These diagnostic algorithms are designed to balance the large number of dementia patients with the varying availability and feasibility of instrumental tools, ensuring applicability across different levels of cognitive impairment—primarily in clinical practice, but also in selected research settings. Particular attention has been given to the selection of questionnaires and scales validated for use in the Italian population.

Achieving the most accurate diagnosis of each sleep and circadian disturbance is essential for guiding appropriate, well‐integrated therapeutic strategies throughout the course of dementia.

## 8 CONCLUSIONS

In the past decades an increasingly close relationship between sleep, sleep–wake rhythm and dementia has been demonstrated and new evidence is growing with possible strategies to prevent dementias also through the management of sleep disorders. As Musiek and Ju wrote “A “tsunami” of AD looms in the coming 20–30 years, and sleep–circadian therapies that reduce AD risk would monumentally benefit societal health” and thus reduce social and economic costs.^262^ Researchers, clinicians, and public authorities should implement collaborative trials in this area to bridge the gap between bench and bedside. A fundamental step in this relevant effort is an accurate and affordable diagnosis of sleep disturbances in different forms of dementia and in different stages of the disease. The approach proposed in this manuscript to diagnose sleep and circadian disturbances in dementia offers a dynamic framework that balances complexity, flexibility, and feasibility in clinical practice, in accordance with the principles outlined in the preceding sections on clinical and technical methods. Although the framework should be adapted to individual case evaluations and to the resources available in each outpatient or clinical setting, its core principles are summarized in the accompanying figure and table. Importantly, the structure is designed to allow progressive refinement and adaptation in response to ongoing technological advances and emerging scientific evidence.

PSG remains the gold standard for the diagnosis of several sleep disorders even in persons with cognitive decline. Questionnaires and rating scales, while practical and easy to administer, still require methodological refinement and contextual adaptation when used with older adults and individuals with cognitive decline. A range of less complex and less costly instrumental tools lies between these two extremes and can support reliable diagnostic processes. Future research should be directed toward establishing an increasingly integrated framework for screening, diagnosis, and treatment of sleep and circadian rhythm disturbances in populations with cognitive decline. Advancing such a framework will be essential to consolidate the evidence base and to enhance the effectiveness of nonpharmacologic and pharmacologic therapeutic interventions tailored to patients but also on caregiver's needs and problems. Developed within the context of the SINdem “Sleep” Study Group, this document is conceived as an expanded and adaptable tool, suitable for use across diverse national settings and capable of supporting multicenter, multidisciplinary, and international collaborations.

## AUTHOR CONTRIBUTIONS

**Biancamaria Guarnieri**: Conceptualization. **Biancamaria Guarnieri**: Methodology. **Biancamaria Guarnieri**: Project administration. **Domeniko Hoxhaj, Francesca Buracchi Torresi**: Software. **Biancamaria Guarnieri, Michelangelo Maestri Tassoni**: Supervision. **Domeniko Hoxhaj, Francesca Buracchi Torresi, Gemma Lombardi**: Visualization. **All authors**: Writing—original draft. **Biancamaria Guarnieri, Michelangelo Maestri Tassoni, Domeniko Hoxhaj, Francesca Buracchi Torresi, Rosalia Silvestri**: Writing—review and editing. All authors reviewed and approved the final version of the manuscript and agree to be accountable for all aspects of the work.

## CONFLICT OF INTEREST STATEMENT

The authors declare no conflicts of interest related to this study. Author disclosures are available in the Supporting Information.

## FUNDING INFORMATION

This research did not receive any specific grant from funding agencies in the public, commercial, or not‐for‐profit sectors.

## Supporting information



Supporting Information: alz71654‐Sup‐0001‐COI disclosures def.pdf

## Data Availability

The data supporting the findings of this study are available from the corresponding author upon reasonable request.
